# Supplementary material for: Long- and short-ranged chiral interactions in DNA-assembled plasmonic chains
Source: Nat Commun. 2021 Apr 1;12:2025. doi: 10.1038/s41467-021-22289-8 (PMC8016906; doi:10.1038/s41467-021-22289-8)
Supplement: Supplementary file 1 — Supplementary Information [file 41467_2021_22289_MOESM1_ESM.pdf]

Supplementary Information

Long- and Short-Ranged Chiral Interactions in DNA-assembled Plasmonic Chains

Kevin Martens[1], Felix Binkowski[2], Linh Nguyen[1], Li Hu[3], Alexander O. Govorov[4], Sven Burger[2, 5] and Tim Liedl[1]*

[1]*Faculty of Physics, Ludwig-Maximilians-University, Geschwister-Scholl-Platz 1, D-80539 Munich, Germany*

[2]*Zuse Institute Berlin, Takustraße 7, D-14195 Berlin, Germany*

[3]*Chongqing Engineering Laboratory for Detection, Control and Integrated System, Chongqing Technology and Business University, Chongqing 400067, China*

[4]*Department of Physics and Astronomy, Nanoscale and Quantum Phenomena Institute, Ohio University, Athens, Ohio 45701, United States*

[5]*JCMwave GmbH, Bolivarallee 22, D-14050 Berlin, Germany*

** tim.liedl@lmu.de*

Supplementary Methods:

DNA scaffold strands (p8064) were prepared following previously described procedures.[1, 2] Unmodified staple strands (purification: desalting) were purchased from Eurofins MWG. Thiol-modified strands (purification: HPLC) were purchased from Biomers. Uranyl formate for negative TEM staining was purchased from Polysciences, Inc.. Spherical gold nanoparticles were purchased from BBI Solutions. Other chemicals were purchased from CarlRoth and Sigma-Aldrich.

Supplementary Note 1: DNA Origami Design

The left half of the dimeric origami structure (yellow part in Figure S1) was folded with an 8064 base pair (bp) scaffold strand and 126 core staple strands as well as 62 "C_4 endcap" staples, 22 staples for dimerization and 12 "handle" strands for NP assembly. The right half of the origami structure (blue part in Figure S1) was folded separately but with the same 8064 bp scaffold strand and a different set of 123 core staple strands as well as 70 C_4 endcap staples, 25 staples for dimerization and 11 handle strands for NP assembly.

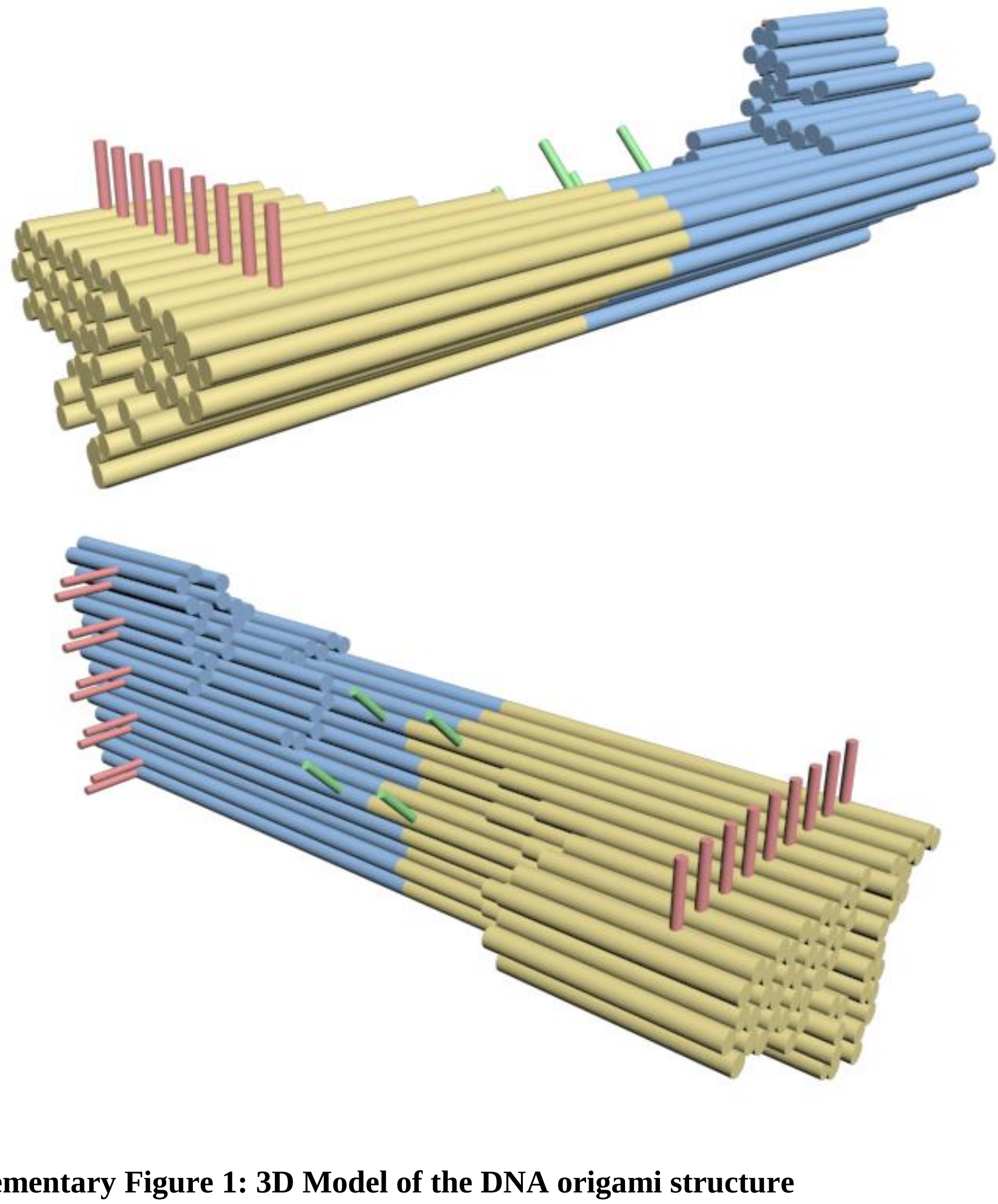

Supplementary Figure 1: 3D Model of the DNA origami structure
The individual halfs are depicted in blue and yellow (cylinders represent DNA helices). The handles for the NS are depicted in green as well as the handles for the NRs in red.

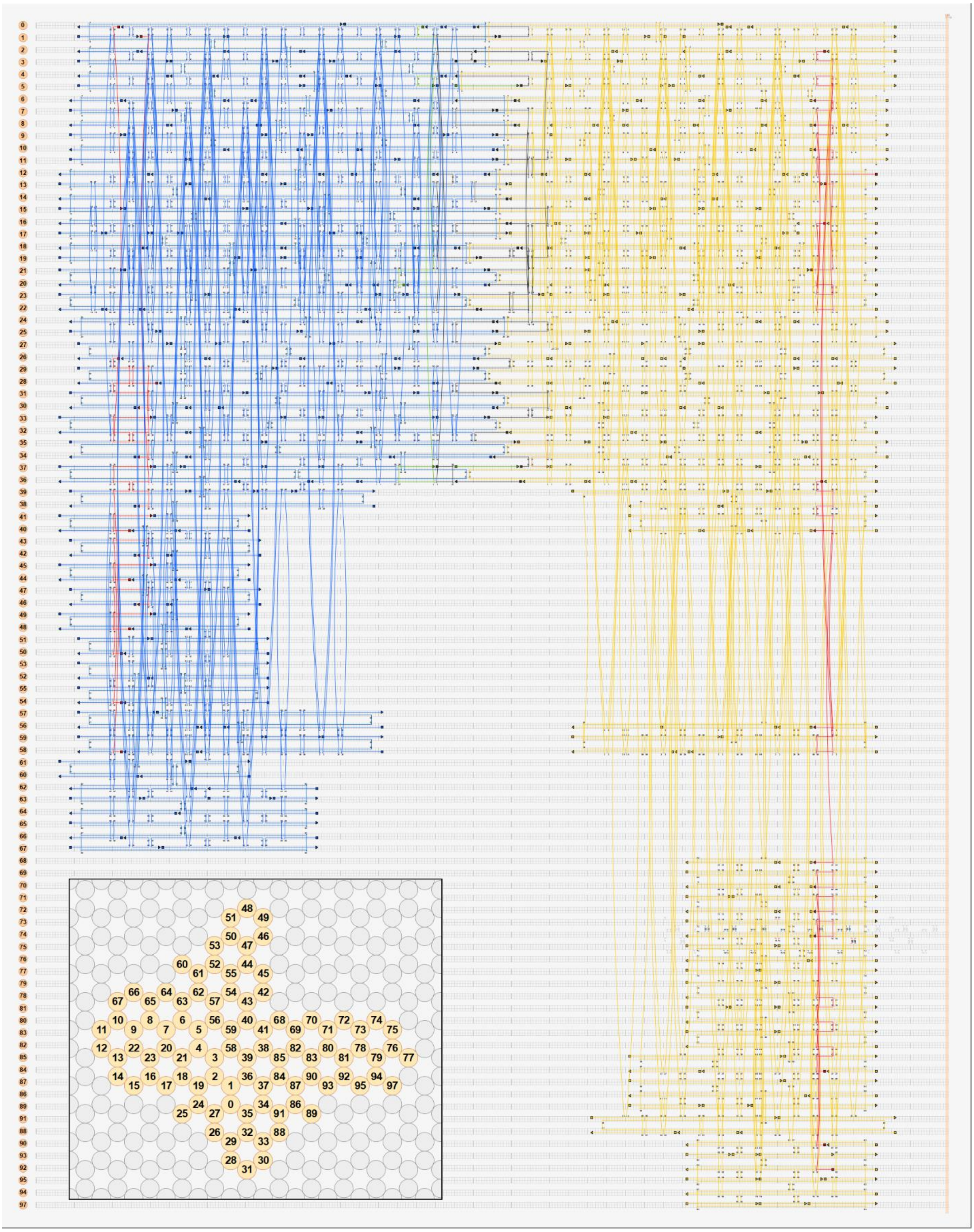

Supplementary Figure 2: Cadnano design of the DNA origami structure

The individual halves are depicted in blue and yellow as well as the handles for the NS in green and the handles for the NRs in red. The staples that connect the individual sides are depicted in black.

Supplementary Note 2: DNA Origami Synthesis

The scaffold was used during folding at a concentration of 16 nM with 160 nM core and endcap staples plus 200 nM dimer and handle staples. 1X TE and 28 mM $MgCl_2$ were added as buffer solution. The two solutions containing the two mixtures were heated up to 65°C and then cooled down to room temperature over the course of 24 h. Subsequently the left side of the origami and the right side were combined in equal amounts and left to dimerize over 48 h. Dimer origamis were purified using gel electrophoresis with 0.7% agarose gel in a buffer of 1X TAE, 11 mM $MgCl_2$ and 0.05‰ Roti Stain as intercalating dye. The gel was run for 2.5 h at 70 V, before the origami dimer band was cut out under UV light and afterwards squeezed to redisperse the sample in buffer.

Supplementary Table 1: Left half DNA origami protocol

**Component**	**Concentration**	**Amount**	**End Concentration**
Scaffold 8064	100 nM	16 µL	16nM
Core Staples	397 nM	40.3 µL	160nM
Endcap Staples	806 nM	19.9 µL	160nM
Dimere Staples	2273 nM	8.8 µL	200nM
Handles	4167 nM	4.8 µL	200nM
TE	20X	5 µL	1X
MgCl2	1 M	2.8 µL	28mM
H2O	-	2.4 µL	-
**Total**	**100 nM**	**100 µL**	

Supplementary Table 2: Right half DNA origami protocol

Component	Concentration	Amount	End Concentration
Scaffold 8064	100 nM	16 µL	16nM
Core Staples	407 nM	39.3 µL	160nM
Endcap Staples	714 nM	22.4 µL	160nM
Dimere Staples	2000 nM	10 µL	200nM
Handles	4545 nM	4.4 µL	200nM
TE	20X	5 µL	1X
MgCl2	1 M	2.8 µL	28mM
H2O	-	0.1 µL	-
**Total**	**100 nM**	**100 µL**	

For TEM analysis, samples were incubated for 15 min on copper grids (Ted Pella Inc., Redding, USA) before being dabbed off with a filter paper and subsequently stained with 2% uranyl format in two steps. In the first step the uranyl format solution only quickly washes the grid, in the second step it is left to incubate for 15 s before being dabbed off. Images were taken with a JEOL JEM 1011 electron microscope at 80 kV.

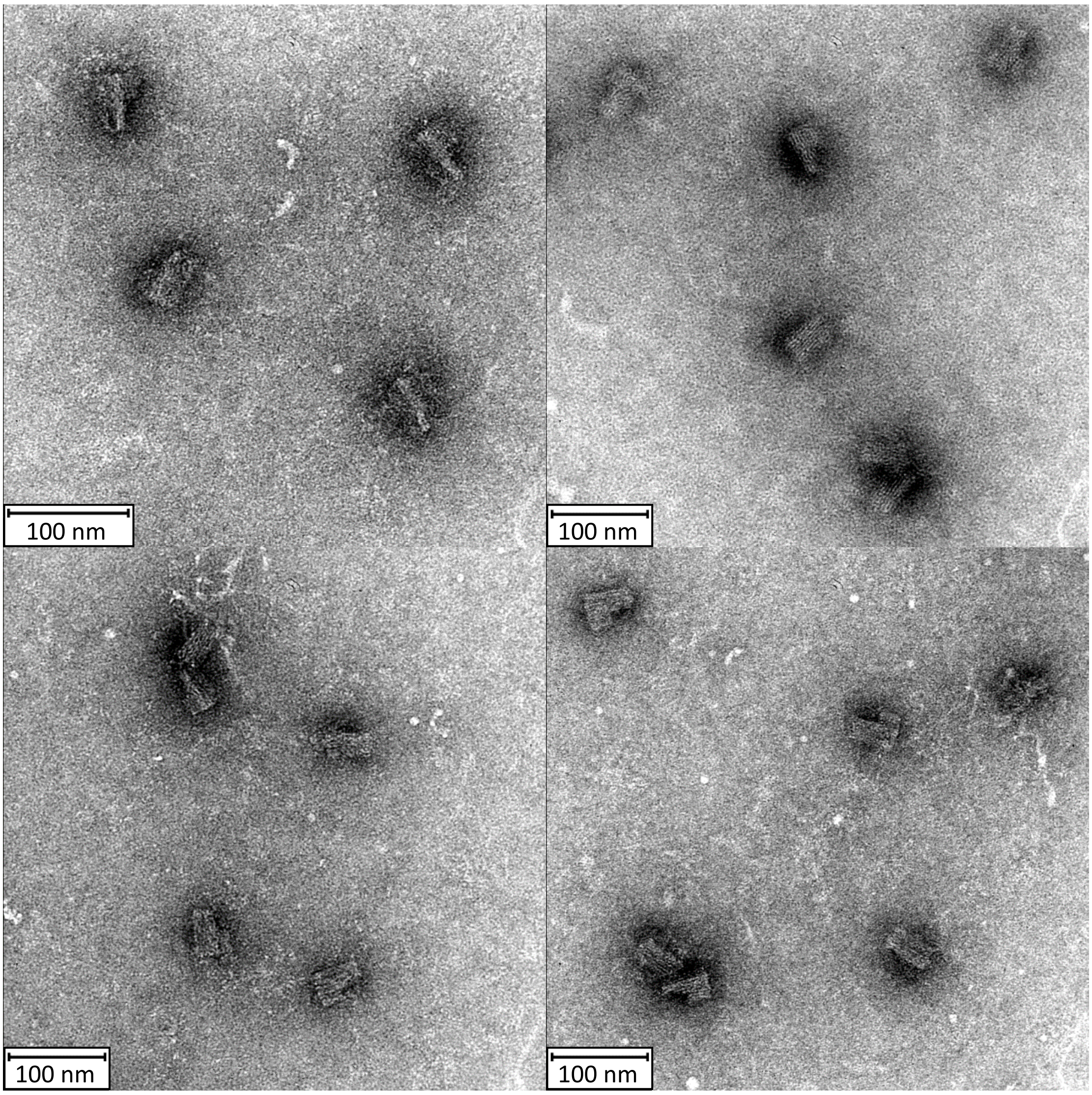

Supplementary Figure 3: Electron micrographs of the left half of the DNA origami

The DNA origami were purified using gel electrophoresis purification and imaged with transmission electron microscopy using uranyl format for staining.

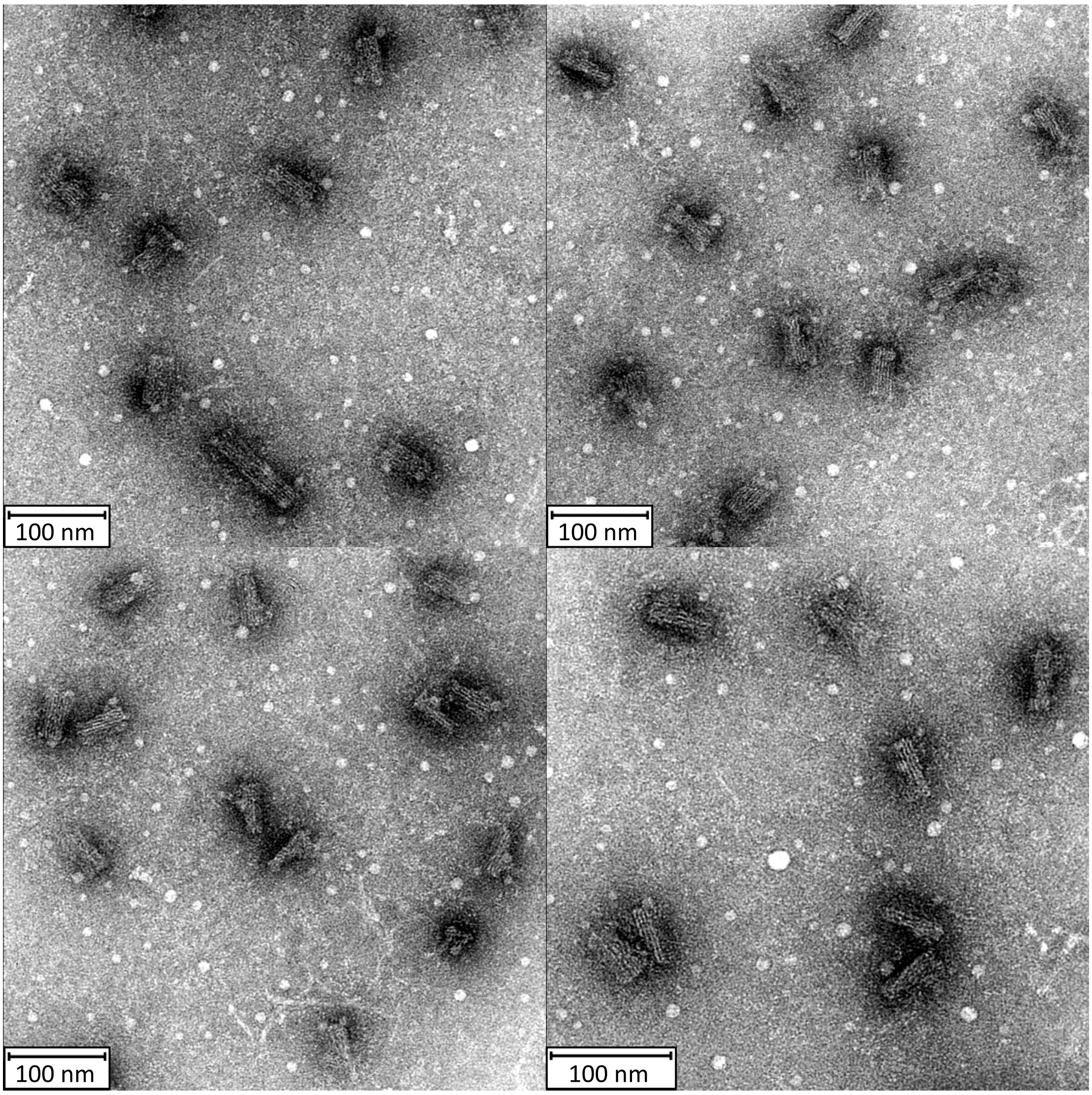

Supplementary Figure 4: Electron micrographs of the right half of the DNA origami

The DNA origami were purified using gel electrophoresis purification and imaged with transmission electron microscopy using uranyl format for staining.

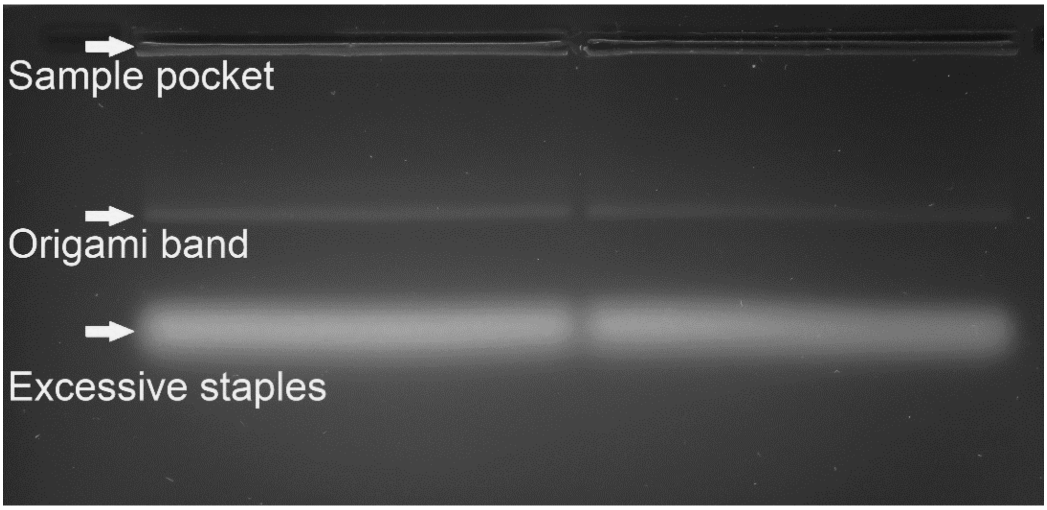

Supplementary Figure 5: DNA origami gel electrophoresis bands

Gel electrophoresis was performed for 2.5 hours at 70 V, showing a band of DNA origami structure after dimerization and a band of excessive staples.

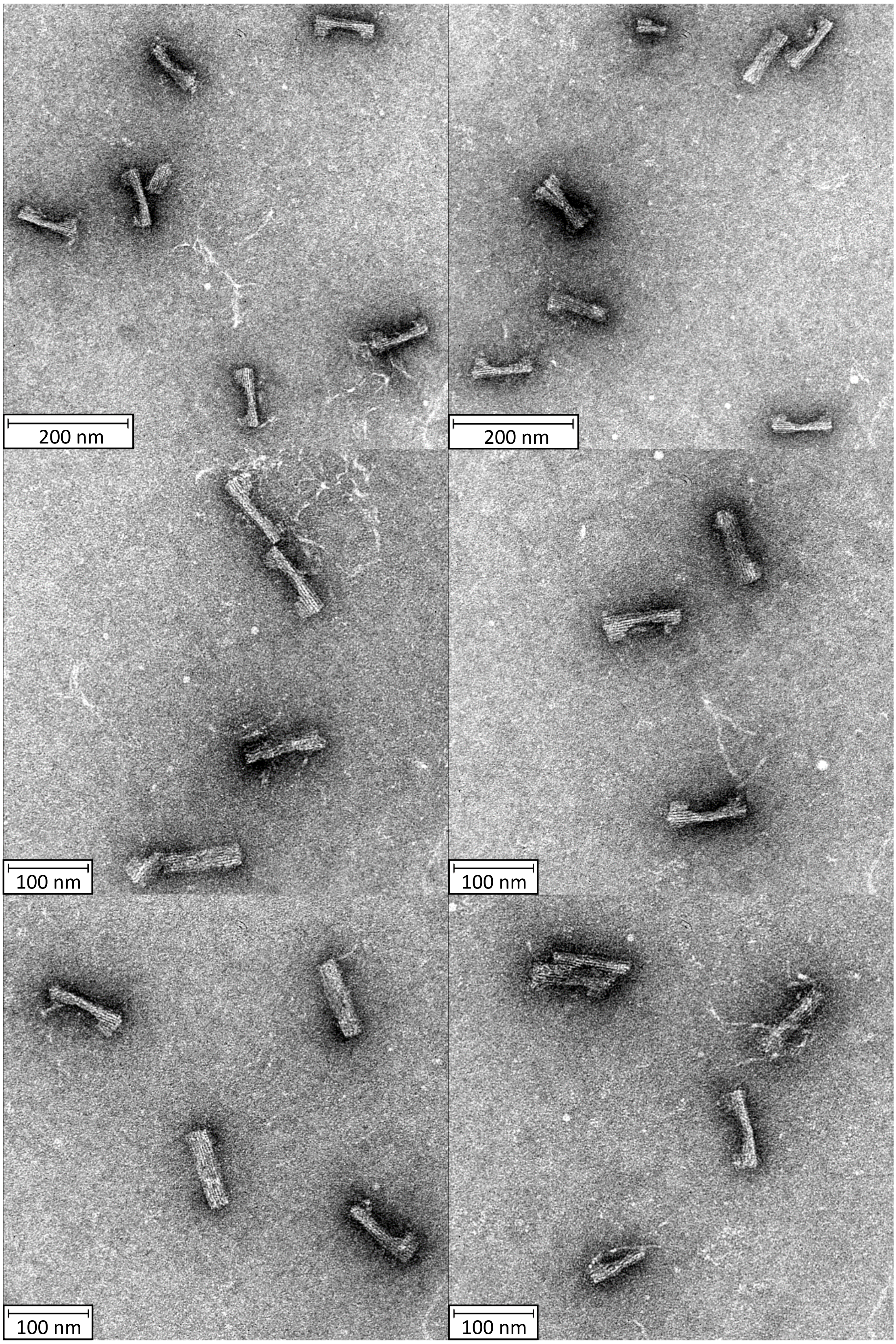

Supplementary Figure 6: Electron micrographs of the complete DNA origami structure

The DNA origami were purified using gel electrophoresis purification and imaged with transmission electron microscopy using uranyl format for staining.

Supplementary Note 3: DNA Origami-Nanoparticles Assembly Synthesis

40 nm NSs were incubated at an optical density (OD) of 4 with 10 mM thiol-modified DNA oligonucleotides, previously activated with TCEP, and 0.02% SDS. NR synthesis was performed following the protocol of Ye et al.[3] 60 nm x 23 nm NRs were incubated at OD 1.4 with 5 mM thiol-modified DNA oligonucleotides and 0.1% SDS. Samples were frozen, thawed and purified using gel electrophoresis with a 0.7% agarose gel in a buffer of 1X TAE, 11 mM $MgCl_2$, run for 1.5 h at 120 V. Subsequently the correct monomer bands were cut and squeezed to redisperse in buffer.

For the synthesis of the NR– –NR sample, NRs were added to the origami structures in a ratio of 10:1 in a buffer of 1X TAE, 11 mM MgCl2 plus 500 mM NaCl and incubated for 24 h. The sample was purified using gel electrophoresis with a 0.7% agarose gel in a buffer of 1X TAE, 11 mM $MgCl_2$, run for 1.5 h at 70 V. The band of the structures was cut out and squeezed. For the NR–NS–NR sample, first NSs were incubated with the DNA origami in a ratio of 5:1 in a buffer of 1X TAE, 11 mM $MgCl_2$ plus 500 mM NaCl for 24 h. Afterwards NRs were added in a ratio of 10:1 to the origami, and incubated in the same buffer for 24 h. The samples were purified using gel electrophoresis with a 0.7% agarose gel in a buffer of 1X TAE, 11 mM $MgCl_2$, run for 1.5 h at 70 V. The structure bands were cut out, squeezed and identified by TEM.

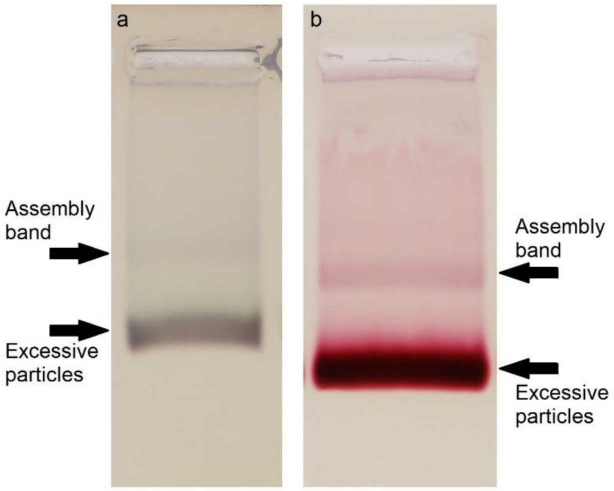

Supplementary Figure 7: Nanostructure gel electrophoresis bands

Bands showing (a) the NR– –NR arrangement and (b) the NR–NS–NR arrangement

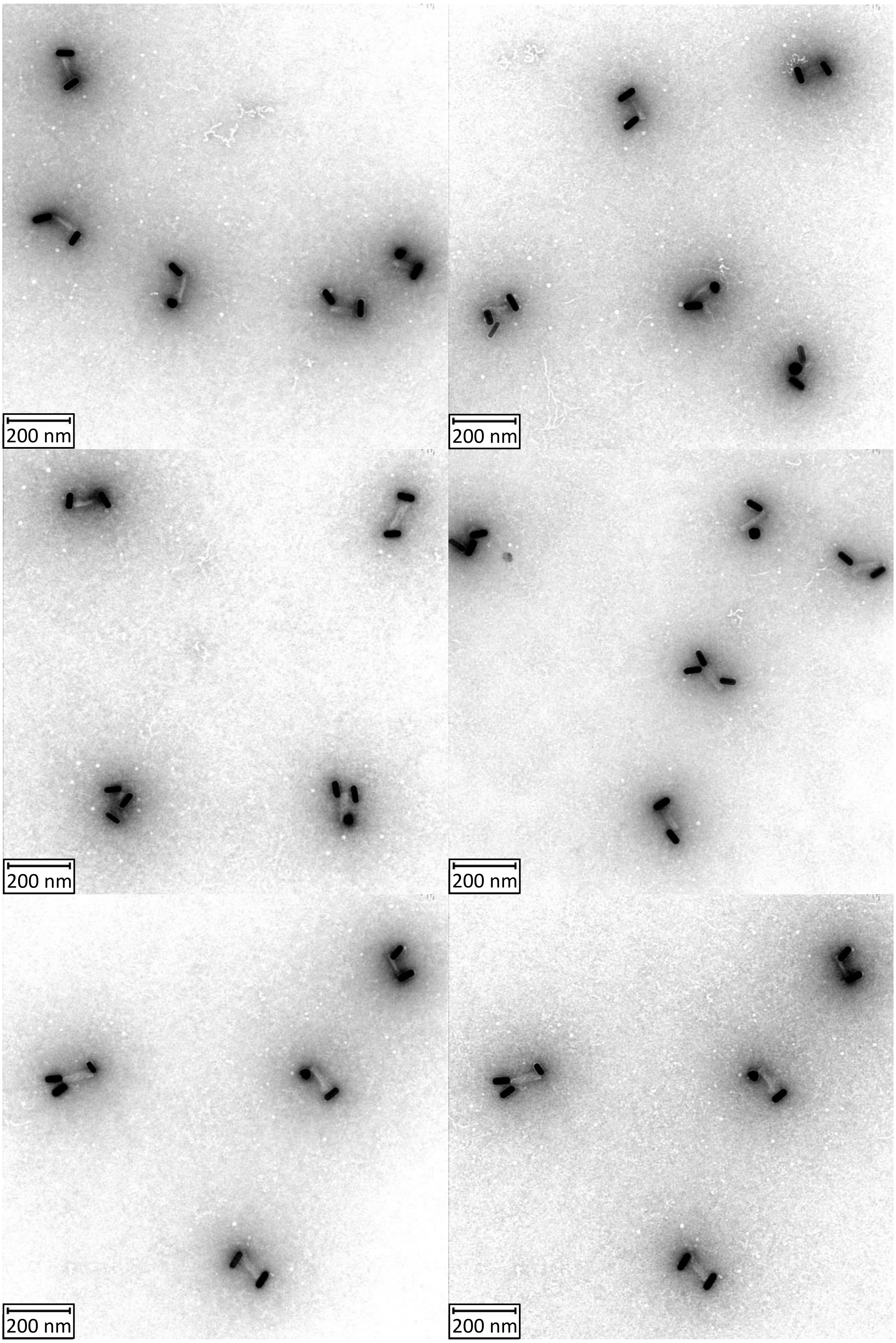

Supplementary Figure 8: Electron micrographs of the NR– –NR arrangement

The DNA origami were purified using gel electrophoresis purification and imaged with transmission electron microscopy using uranyl format for staining.

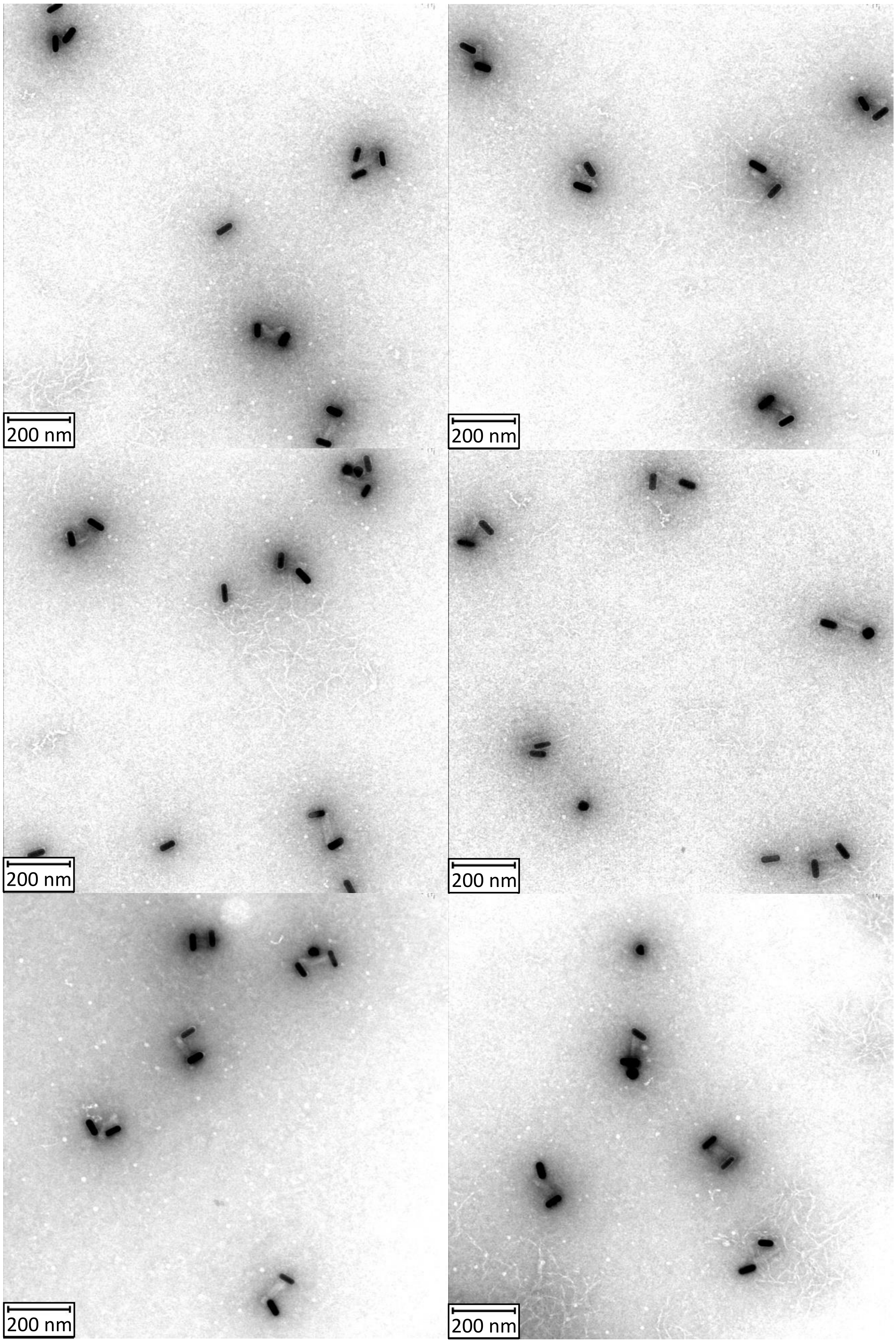

Supplementary Figure 9: Electron micrographs of the NR– –NR arrangement

The DNA origami were purified using gel electrophoresis purification and imaged with transmission electron microscopy using uranyl format for staining.

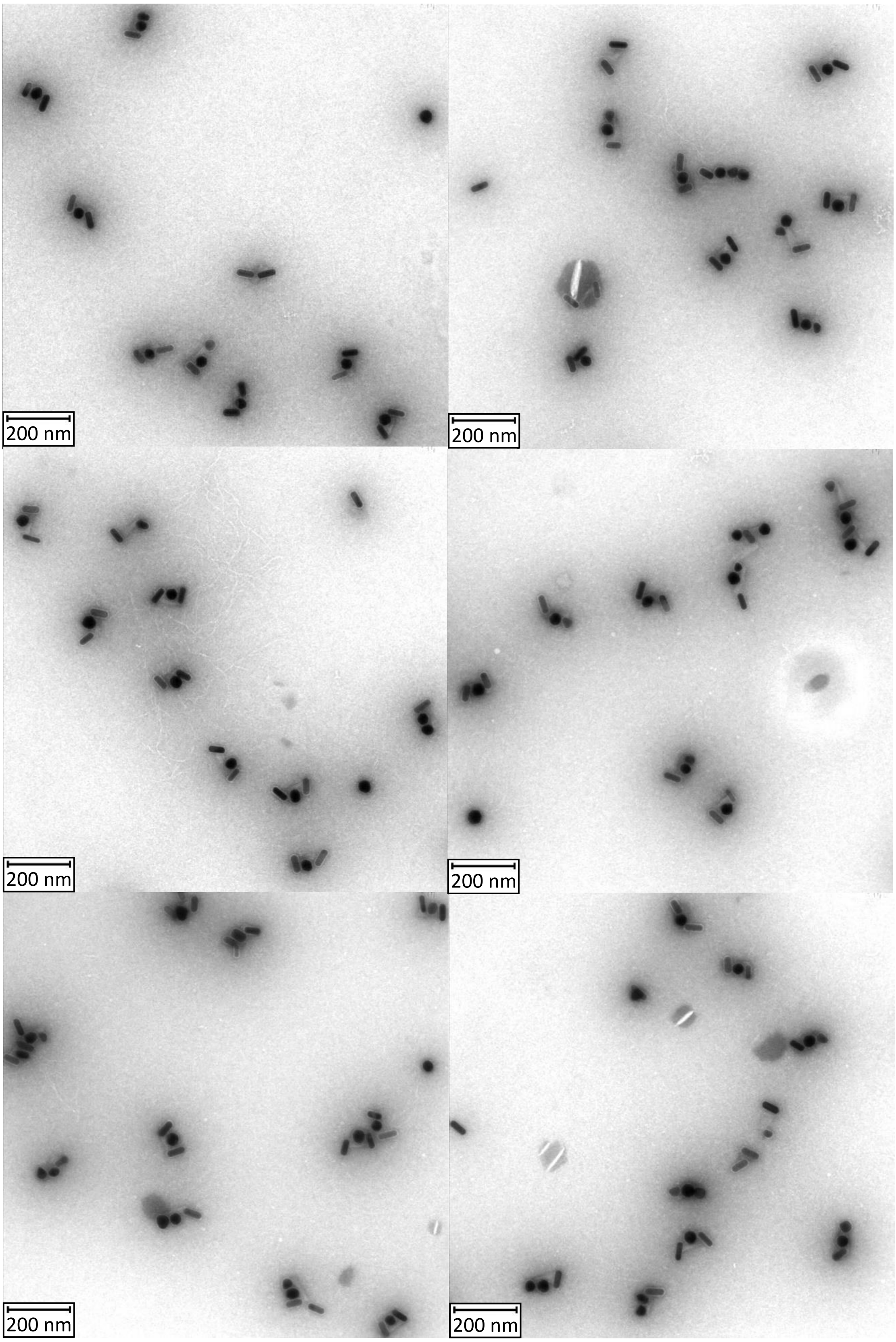

Supplementary Figure 10: Electron micrographs of NR–NS–NR sample

The DNA origami were purified using gel electrophoresis purification and imaged with transmission electron microscopy using uranyl format for staining.

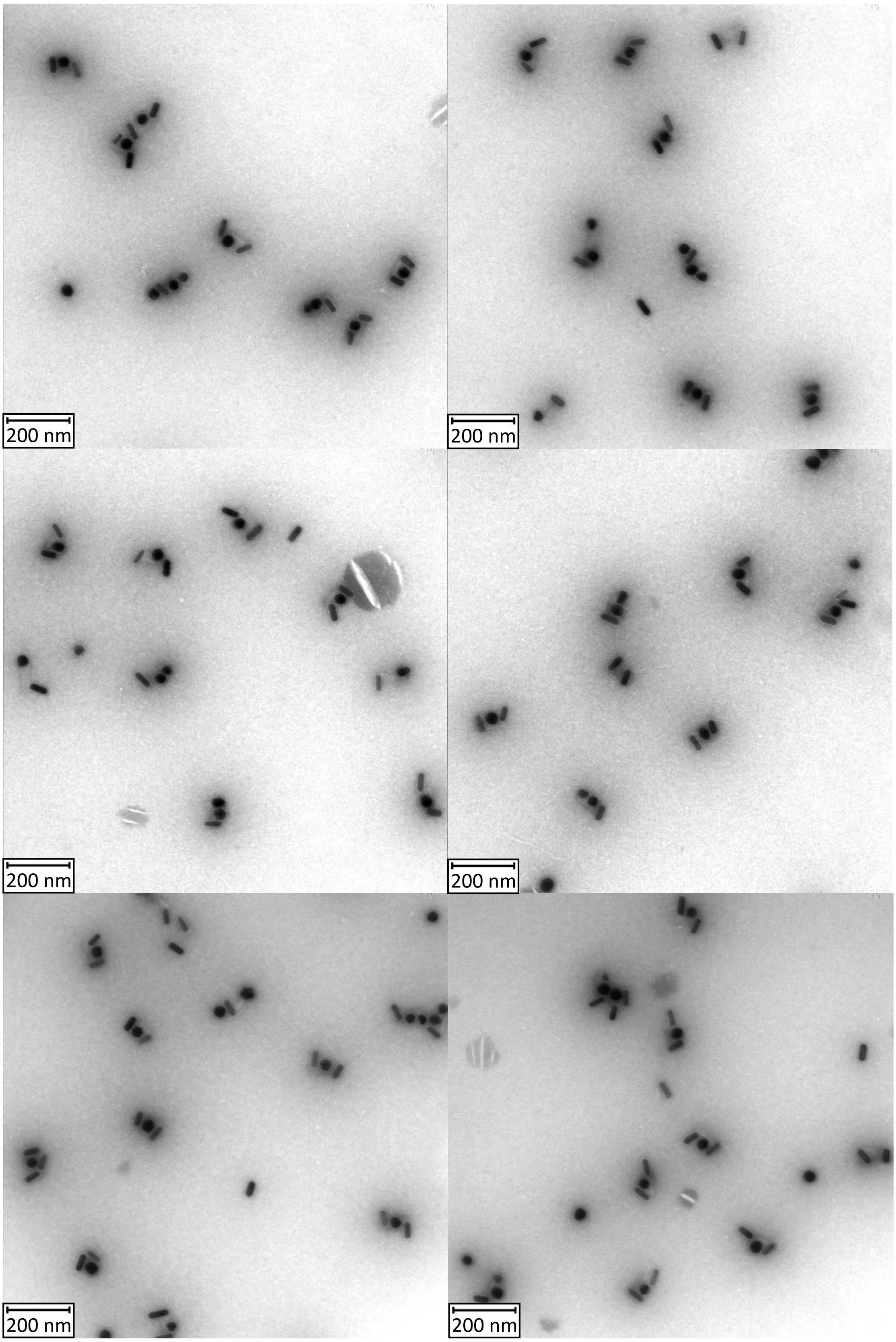

Supplementary Figure 11: Electron micrographs of NR–NS–NR sample

The DNA origami were purified using gel electrophoresis purification and imaged with transmission electron microscopy using uranyl format for staining.

Supplementary Table 3: NR– –NR Synthesis Assembly Statistics

**Assembly**	**Number**	**Percentage**
NR– –	3	< 3 %
NR– –NR	113	57.5 ±7 %
NS– –NR	27	14 ±4.5 %
NS– –NS	1	< 1.5 %
NR– –2NR	30	15.5 ±5 %
NR–NS–NR	7	3.5 ±2.5 %
NR–NR–NS	8	4 ±2.5 %
NR–NS–NS	3	< 3 %
NS–NR–NS	1	< 1.5 %
NR–NR–NR–NR	2	< 2 %
NR–NR–NS–NS	1	< 1.5 %
**Total**	**196**	**100 %**

Supplementary Table 4: NR– –NR Synthesis Particle Statistics

**Particle**	**Number**	**Percentage**
NS	54	12 ±3 %
NR	390	88 ±3 %
**Total**	**444**	**100 %**

Supplementary Table 5: NR–NS–NR Synthesis Assembly Statistics

**Assembly**	**Number**	**Percentage**
NR– –NR	28	9 ±3 %
NS– –NR	11	3.5 ±2 %
NS– –NS	3	< 2 %
NR– –2NR	4	1.5 ±1 %
NR–NS–NR	160	51 ±5.5 %
NS– –2NR	8	2.5 ±1.5 %
NR–NS–NS	57	18 ±4.5 %
NS–NR–NS	4	1.5 ±1 %
NS–NS–NS	5	1.5 ±1.5 %
NR–2NS–NR	5	1.5 ±1.5 %
NR–NR–2NR	3	< 2 %
NS–NS–2NR	3	< 2 %
NR–NS–NR–NS	7	2 ±1.5 %
NR–NS–2NR	7	2 ±1.5 %
NR–NS–2NS	4	1.5 ±1 %
NS–NR–2NS	3	< 2 %
NS–NR–3NS	1	< 0.5 %
**Total**	**313**	**100 %**

Supplementary Table 6: NR– –NR Synthesis Particle Statistics

**Particle**	**Number**	**Percentage**
NS	354	41 ±3 %
NR	577	59 ±3 %
**Total**	**931**	**100 %**

Supplementary Note 4: CD and Extinction Measurements

Samples were measured with a Chirascan circular dichroism spectrometer (Applied Photophysics, Surrey, UK) in cuvettes with 3 mm pathlengths. Spectra were collected in 0.5 nm steps with 0.3 s for each step. 3 measurements were made and averaged for the NR– –NR sample.

Supplementary Note 5: Numerical Simulations

<u>Numerical methods</u>

For solving Maxwell's equations, we use JCMsuite, version 3.18. To ensure high numerical accuracy, we choose a polynomial degree of the FEM ansatz functions of p=2 and a mesh element edge size smaller than 7 nm for the NPs and 14 nm for background material. Transparent boundary conditions are realized by using perfectly matched layers.

The absorbed electromagnetic field energy is obtained through volume integration of the electromagnetic field energy density in the various physical objects. The scattered electromagnetic field energy is obtained through surface integration of the electromagnetic energy flux density over the boundary of the computational domain. Field patterns for visualization purposes (Fig. 3e, f) are obtained by exporting the computed near fields on specific cross-sections and a summation of the exported fields over all source terms at a specific wavelength.

For performing numerical parameter studies as shown in Fig. 4 and Fig. S12, the physical quantities of the project are parameterized, and a scripting language (Matlab) is used to automatically generate the input files and to distribute the FEM computations to various threads on a workstation for parallel computation of the parameter- and wavelength-scans.

Numerical study to investigate impact of finite particle placement accuracy

In order to address the question of the impact of NP particle placement accuracy on the obtained g factor of the NP arrangement, we have investigated the impact of the relative displacement of the NRs over a large parameter range. Here we displace the NRs relative to the design values by -6 nm to 75nm in the same way as shown in Fig. 4a.

Figure S12 (a) shows several selected CD spectra for different displacement values. The maximum value of the CD spectrum was extracted for each rod displacement and the result is plotted for the various relative rod displacements in Figure S12 (b). It can be seen that the CD response increases with rod displacement until a maximum value is obtained at ~14nm. For larger displacements, the normalized CD response decreases. The sensitivity of the obtained CD response on rod displacement is quite high for low displacements, however, at the design with maximum value, the sensitivity with respect to rod displacement is vanishing. This study demonstrates that it is possible to use numerical design for proposing setups with low sensitivity with respect to parameter variations.

From experimental studies we deduced that the spatial fabrication accuracy for the used DNA origami assembly method is better than 1 nm.[4] This implies, in our case, with a rod displacement of 0 nm (NRP 4 in Figure 4) that the maximum chiral response is impacted by placement inaccuracy yielding a relative error of maximum CD of below 8%.

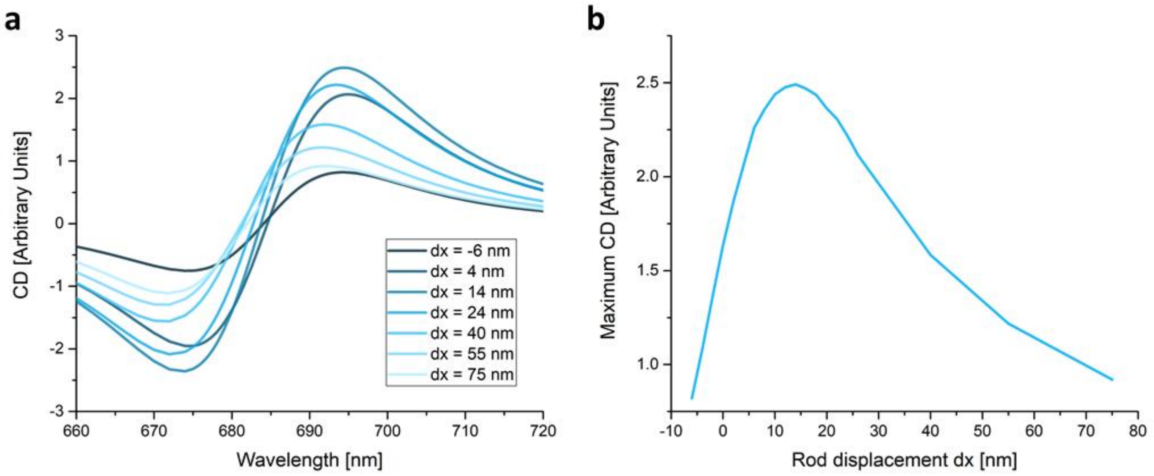

Supplementary Figure 12: **Effect of NR displacement**

(a) CD spectra for varying dx displacement. (b) Maximum CD response for relative rod displacement in NR–NS–NR assemblies. Notably, around displacement of 14 nm, there is a broad maximum at which the signal sensitivity with respect to dx is vanishing. This study demonstrates that it is possible to use numerical design for proposing setups with low sensitivity with respect to parameter variations.

Numerical study to investigate far-field effects

To study the impact of far-field coupling between the NPs we have performed numerical simulations where we vary the interparticle distance (center-to-center distance, in y-direction, corresponding to the axis of NP arrangement). Here, we use both, the NR-NS-NR setting and the NR--NR setting. Again, we compute absorption and CD spectra and extract the maximum CD value, normalized to maximum extinction. Fig. S13 shows, for both settings, that this value decreases to negligible values for interparticle distances larger than 100 nm. In contrast to planar nanostructures where the far-field interference effects can be noticeable,[5] our simulations for structures dispersed in solution revealed no relevant far-field effects for a wide range of distances between the NRs.

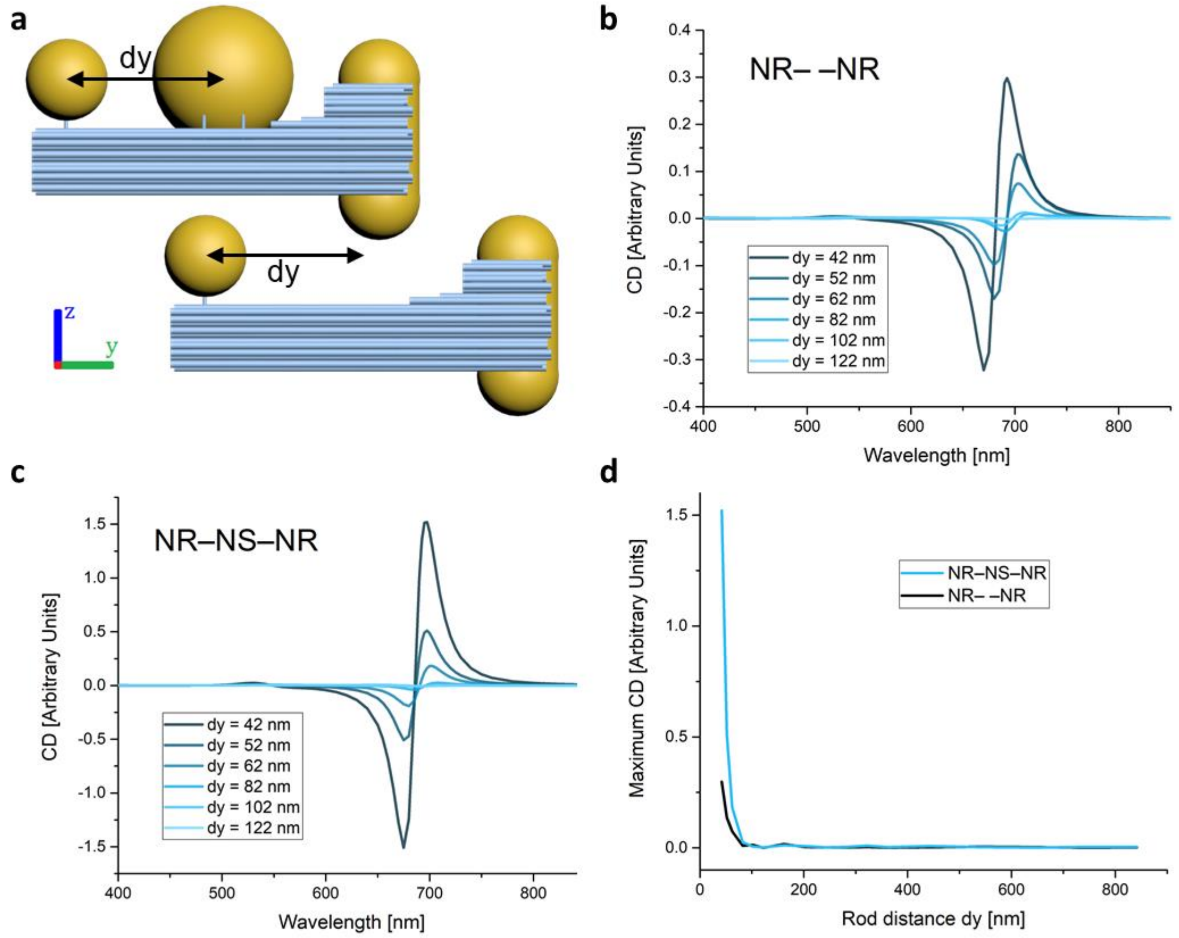

Supplementary Figure 13: Simulated CD as a function of NR displacement

The NRs are displaced in y-direction. (a) Both NRs were moved from their original positions – each 42 nm away from the central NS (center-to-center distance) – up to 840 nm to the left and right, respectively. (b) CD spectra of the chiral structure without the NS in place for varying dy displacements. (c) CD spectra of the chiral structure with the NS for varying dy displacements. (d) Maximum CD responses for relative rod displacement in y-direction in a displacement range from 42 nm to 840 nm. It becomes apparent, that far-field effects are negligible, compared to the strong, hot spot mediated, near-field coupling.

Supplementary References

1. Douglas, S. M. et al. Self-assembly of DNA into nanoscale three-dimensional shapes. *Nature* **459**, 414-418 (2009).
2. Douglas, S. M., Chou, J. J. & Shih, W. M. DNA-nanotube-induced alignment of membrane proteins for NMR structure determination. *PNAS* **104**, 6644-6648 (2007).
3. Ye, X. et al. Improved size-tunable synthesis of monodisperse gold nanorods through the use of aromatic additives. *ACS Nano* **6**, 2804-2817 (2012).
4. Hartl, C. et al. Position accuracy of gold nanoparticles on DNA origami structures studied with small-angle X-ray scattering. *Nano Lett.* **18**, 2609-2615 (2018).
5. Tang, Y., Sun, L., & Cohen, A. E. Chiroptical hot spots in twisted nanowire plasmonic oscillators. *Appl. Phys. Lett.* **102**, 043103 (2013)
