# Supplementary material for: Long- and short-ranged chiral interactions in DNA-assembled plasmonic chains
Source: Nat Commun. 2021 Apr 1;12:2025. doi: 10.1038/s41467-021-22289-8 (PMC8016906; doi:10.1038/s41467-021-22289-8)
Supplement: Supplementary file 4 — Description of Additional Supplementary Files [file 41467_2021_22289_MOESM4_ESM.pdf]

Description of Additional Supplementary Files

File: Supplementary Data 1

Description: This folder contains files for performing numerical simulations for computing the chiroptical response of metal nanoparticle arrangements. It also contains a readme.txt file explaining all procedures in detail.
